# Surrounding Residential Greenness and Health: Associations With Abdominal Obesity and Dyslipidemia. A Meta-Analysis of Cross-Sectional Studies

**DOI:** 10.3389/phrs.2025.1608163

**Published:** 2025-02-18

**Authors:** Irene Marcilla-Toribio, Bruno Bizzozero-Peroni, Blanca Notario-Pacheco, Shkelzen Cekrezi, Martin Fernandez-Perez, Ana Perez-Moreno, Ana Diez-Fernandez, Maria Martinez-Andres

**Affiliations:** ^1^ Health and Social Research Centre, Universidad de Castilla-La Mancha, Cuenca, Spain; ^2^ Research Group Health, Gender, and Social Determinants, Instituto de Investigación Sanitaria de Castilla-La Mancha (IDISCAM), Cuenca, Spain; ^3^ Faculty of Nursing, Universidad de Castilla-La Mancha, Albacete, Spain; ^4^ Higher Institute of Physical Education, Universidad de la República, Rivera, Uruguay; ^5^ Faculty of Nursing, Universidad de Castilla-La Mancha, Cuenca, Spain; ^6^ Fundación Biomédica Galicia Sur, Grupo I-Saúde, Vigo, Spain

**Keywords:** green spaces, residential greenness, cholesterol, dyslipidemia, meta-analysis

## Abstract

**Objectives:**

We aimed to quantify the relationships of surrounding residential greenness with abdominal obesity and dyslipidemia.

**Methods:**

A systematic search was conducted in January 2024 through 5 electronic databases including Pubmed, Scopus, Web of Science, and CINHAL (Complete and GreenFILE). The DerSimonian and Laird method was used to calculate the pooled odds ratios (ORs) and their respective 95% confidence intervals (CIs). The study protocol was registered in PROSPERO (CRD42024528548).

**Results:**

Eleven cross-sectional studies involving 564,254 individuals with a mean age of 55.95 years were included. A significant inverse relationship was observed between increased surrounding greenness and lower odds of abdominal obesity (OR: 0.80; 95% CI: 0.70–0.91), elevated TG (OR: 0.97; 95% CI: 0.96–0.97), and low HDL-C levels (OR: 0.98; 95% CI: 0.95–1.00).

**Conclusion:**

Abdominal obesity and elevated triglyceride levels could be reduced in the general adult population by increasing residential greenness. These findings underscore the importance of integrating greenness into urban planning and public health policies to promote healthier environments. Interventions such as the development of urban green spaces could play a crucial role in reducing cardiometabolic risk factors.

**Systematic Review Registration:**

https://www.crd.york.ac.uk/prospero/display_record.php?ID=CRD42024528548

## Introduction

Urbanization is rapidly increasing globally. Currently, over half of the worldwide population resides in urban environments, a figure projected to increase to 60% by 2030 [1–3]. This phenomenon has resulted in a significant increase in the number of individuals residing in areas characterized by air pollution, noise, and a reduction in green space [4–6]. Indeed, changes in urban design have been suggested with a focus on the concept of “healthy cities” to improve population health. Green spaces are one of the important pillars of this new and innovative approach [7, 8], constituting a fundamental component of the Sustainable Development Goals outlined within the World Health Organization’s 2030 Agenda [9].

The urban environment has been identified as a key health determinant as its influence on human health [10], improving outcomes in mental health, maternal and child health and cardiovascular health between others [11–13]. Adults are particularly impacted by urban environments context, as they have the agency to choose how and whether to engage with green spaces. The interaction in this context underscores the significance of study this population to better understand how these spaces can promote health and wellbeing [11, 12]. Moreover, it seems that women may benefit slightly more from greenery than men [14, 15]. In addition, socioeconomic status plays a key role in the relationship between urban greenness and health benefits. Previous studies have indicated that people with higher socioeconomic status tend to have greater access to green spaces, which may favors healthier lifestyles, such as greater physical activity levels [16–18]. However, proximity to urban vegetation may also be particularly beneficial for individuals from lower socioeconomic backgrounds, who often face higher cardiovascular risk due to inequalities in access to health resources and less favorable lifestyles [17–19].

Cardiovascular disease (CVD) represents a significant global public health concern. In 2019, circulatory system diseases were the leading cause of mortality worldwide, accounting for 32% of all deaths [20]. Additionally, it is estimated that by 2030, the number of deaths from cardiocirculatory disease will reach 23.6 million per year [20]. Risk factors for CVD include hypertension, smoking, diabetes, obesity, physical inactivity, and unhealthy diets [21, 22]. Adiposity, in particular, is a significant risk factor of the development of this condition [23, 24] which can be objective and easily quantifiable by waist circumference and blood lipids parameters such as total cholesterol (TC), triglycerides (TG), low-density lipoprotein cholesterol (LDL-C), and high-density lipoprotein cholesterol (HDL-C) [25–28].

Residential greenness can be objectively measured. The Normalized Difference Vegetation Index (NDVI) is a widely used numeric measure of vegetation which is effective in quantifying its health and density [29]. This index has been used in several studies related to public health, as a greater presence of green areas has been associated with benefits for people’s health [11, 12, 30]. The NDVI’s widespread use is due to its ability to provide objective, reliable and easily interpretable information on the state of vegetation, making it a valuable and indispensable tool for both ecological monitoring and health research [30, 31].

The association between green spaces and obesity has been studied. However, the main focus has been on body mass index (BMI) or CVD mortality, and the findings are inconclusive regarding the amount and type of green space needed to reach health benefits [32]. Consequently, it is imperative to acknowledge that there are still aspects that require further investigation. Particularly, studies focusing on waist circumference and blood lipids levels, which are a more specific marker of body fat distribution and CVD risk factors [33, 34].

Our study is the first meta-analysis to examine the relationship between proximity to urban greenness and its specific influence on waist circumference and individual blood lipid markers (cholesterol, HDL, LDL, and triglycerides) in adults. While previous research has highlighted the general health benefits of urban greenness, no study has comprehensively quantified its associations with these cardiometabolic risk factors. By addressing this gap, our findings provide novel insights into the potential mechanisms through which urban green spaces can support chronic disease prevention and cardiovascular mortality risk reduction.

Therefore, the main aim of this systematic review and meta-analysis was to quantify the relationships of surrounding residential greenness with abdominal obesity and dyslipidemia in adults. In addition, the possible influence of sociodemographic variables, such as sex and socioeconomic status, on this relationship was also explored.

## Methods

### Design

This systematic review and meta-analysis was conducted according to the Meta-analysis of Observational Studies in Epidemiology (MOOSE) statement [35] and was performed following the recommendations of the Cochrane Collaboration Handbook [36]. The study protocol was registered in PROSPERO (CRD42024528548).

### Search Strategy

A systematic search for studies was conducted through 5 electronic databases including Pubmed, Scopus, Web of Science, and CINHAL (Complete and GreenFILE). The search was conducted from inception until 19th January 2024. The following free terms combined with Boolean operators were used following the PECO (Population, Exposure, Comparison, Outcome) strategy [37]: [(“adults”) AND (“greenness” OR “green space” OR greenery OR “residential greenness” OR “urbanization” OR neighborhood OR “NDVI”) AND (“waist circumference” OR “abdominal obesity” OR “cardiometabolic risk” OR adiposity OR “body fat” OR “body composition” OR “fat mass” OR “lean mass” OR “visceral fat” OR “cholesterol” OR “blood lipid”)]. No filters were used. No temporal restrictions were applied during the search of the literature.

In the course of the identification, selection and exclusion of relevant articles, the Zotero software [38] was employed for the management of bibliographic references. This software facilitated the efficient organization of references obtained through literature searches, as well as the review and selection of articles. Zotero also assisted in the management of citations and the maintenance of a clear record of the articles included and excluded at each stage of the process, which was essential to ensure the transparency and reproducibility of the methodology.

### Selection Criteria and Data Extraction

In this systematic review and meta-analysis, two independent researchers (IM-T and MM-A) screened the articles by title and abstract before coming to a consensus opinion to determine whether studies should be included. To be included, the retrieved studies from the peer-reviewed literature had to meet the following inclusion criteria: i) Population: General adult population; ii) Exposure: Residential greenness measured from each participant’s postal address using the NDVI index as continuous data to avoid heterogeneity introduced by categorization [36]; iii) Outcome: Studies that investigated waist circumference as adiposity parameter, and TC, TG, LDL-C, and HDL-C as blood lipid parameters, using consistent and comparable units of measurement; iv) Design: cross-sectional studies were included. Conference abstracts, commentaries, editorials, or dissertations were excluded. Furthermore, articles written in languages other than English or Spanish were excluded based on the authors’ language proficiency and the restricted availability of translation resources. In the event of any discrepancies, the eligibility of studies were resolved by consensus or with the involvement of a third researcher (B.B-P).

The information extracted from the selected articles was the following: authors and year of publication, country in which data were collected, sample size, female percentage, mean age, mean BMI, NDVI exposure definition, NDVI increment unit, NDVI mean, adiposity and dyslipidemia outcomes, outcome units, outcome cut-off points, statistics including odds ratios (95% CI), and covariate adjustments.

### Exposure

NDVI [39]: this index is employed to measure and monitor vegetation on the earth’s surface by analyzing satellite images. It is based on the difference between the red and near-infrared bands of the electromagnetic spectrum, taking advantage of the fact that vegetation strongly absorbs red light for photosynthesis and reflects near-infrared light. The NDVI index takes values between −1 and 1. Positive values indicate the presence of vegetation, with values close to 1 indicating dense and healthy vegetation. Conversely, negative values or values close to zero indicate the absence of vegetation or non-vegetated surfaces such as water, snow, or bare soil [40].

### Outcomes

Waist circumference [27]: this measurement is an anthropometric assessment employed to evaluate the distribution of adipose tissue and the risk of metabolic disorders, including cardiovascular disease. This measure also is designed to indicate abdominal obesity, and particularly, it appears to be a more specific marker of body fat distribution, in comparison to body mass index [41]. The waist circumference is typically taken around the narrowest portion of the torso, typically situated just above the navel. In addition, waist and hip circumferences can be used to calculate another anthropometric measure of adiposity, the waist-hip ratio [42]. These outcomes were analyzed as categorical data in accordance with the cut-off point employed by each study to define an increased risk of abdominal obesity.

Blood lipids [43]: these are fat molecules that circulate in the bloodstream. An excess of these molecules can accumulate on the walls of arteries, forming plaques. These plaques can impede blood flow and increase the risk of cardiovascular events. The main types of blood lipids include TC, TG, LDL-C and HDL-C, and if one or more of the values established as appropriate exceeds the threshold, dyslipidemia is considered to be present [44, 45]. The blood lipid outcomes were analyzed as categorical variables according to the cut-off points employed by the included studies to define high cholesterol, triglycerides and LDL-C levels, and low HDL-C levels.

To determine the socio-economic status of participants, an approach based on their reported monthly income was used.

### Methodological Quality Assessment

The Quality Assessment tool for Observational Cohort and Cross-Sectional studies from the National Heart, Lung and Blood Institute [46] was employed to assess the methodological quality of the included studies. The assessment of methodological criteria was conducted regarding the following domains: quality of the research question, reporting of the population definition, participation rate, recruitment, sample size, appropriateness of statistical analyses, timeframe for associations, exposure levels, ascertainment of the exposure, appropriateness of the outcome measured, outcome blinding of researchers, loss to follow-up, and confounding variables. The quality assessment of the included articles was evaluated by two independent researchers (I.M-T and M.M-A). Any discrepancies were resolved through consensus or, if necessary, with the involvement of a third researcher (B.B-P).

### Data Analysis

Odds ratios (ORs) and their respective 95% confidence intervals (95% CI) were extracted from the fully adjusted model to estimate the associations between increased NVDI values and the odds of adverse adiposity and blood lipid parameters. This meta-analysis applied a random-effects model to combine the ORs from the included studies and the DerSimonian and Laird method [47] was used to compute the between-study variance. Additionally, a prediction interval for the overall effect was calculated based on the t-distribution with 4 degrees of freedom. This interval provides an estimate of the range within which the true effect size of a new study is expected to fall, accounting for both within-study and between-study variability [48]. Heterogeneity was also examined using the I^2^ statistic, which ranges from 0% to 100%. Based on the I^2^ values, heterogeneity was classified as having no important effect (0%–30%), a moderate effect (30%–60%), a substantial (60%–75%) effect, or a considerable effect (75%–100%) [49]. The p values were also considered for the assessment of heterogeneity (when p < 0.05, heterogeneity was identified).

Other methodological considerations for data collection and analysis should be detailed. In order to prevent over-representation, the pooled OR was estimated in those studies that provided multiple data points per residential buffers [50–55]. Subsequently, a single data set was utilized for each study exhibiting this specificity. In studies in which β coefficient and its CI were reported [56] the OR (95% CI) was estimated by calculating the exponential of β. Furthermore, in studies that provided results on the odds of adverse outcomes according to higher NDVI values only graphically [56, 57], data were extracted using WebPlotDigitizer software [58].

Subgroup analyses were conducted according to sex (male and female) and socioeconomic status (lower and upper), if possible. Residential buffer zones (100 m, 250 m, 300 m, 400 m, 500 m, 800 m, 1,000 m, 1,500 m, and 1,250 m) analyses were also performed.

All statistical analyses were conducted using R software (Foundation for Statistical Computing, V.2023.12.1 + 402, Boston, MA, United States) [59].

## Results

### Baseline Characteristics

**FIGURE 1:**
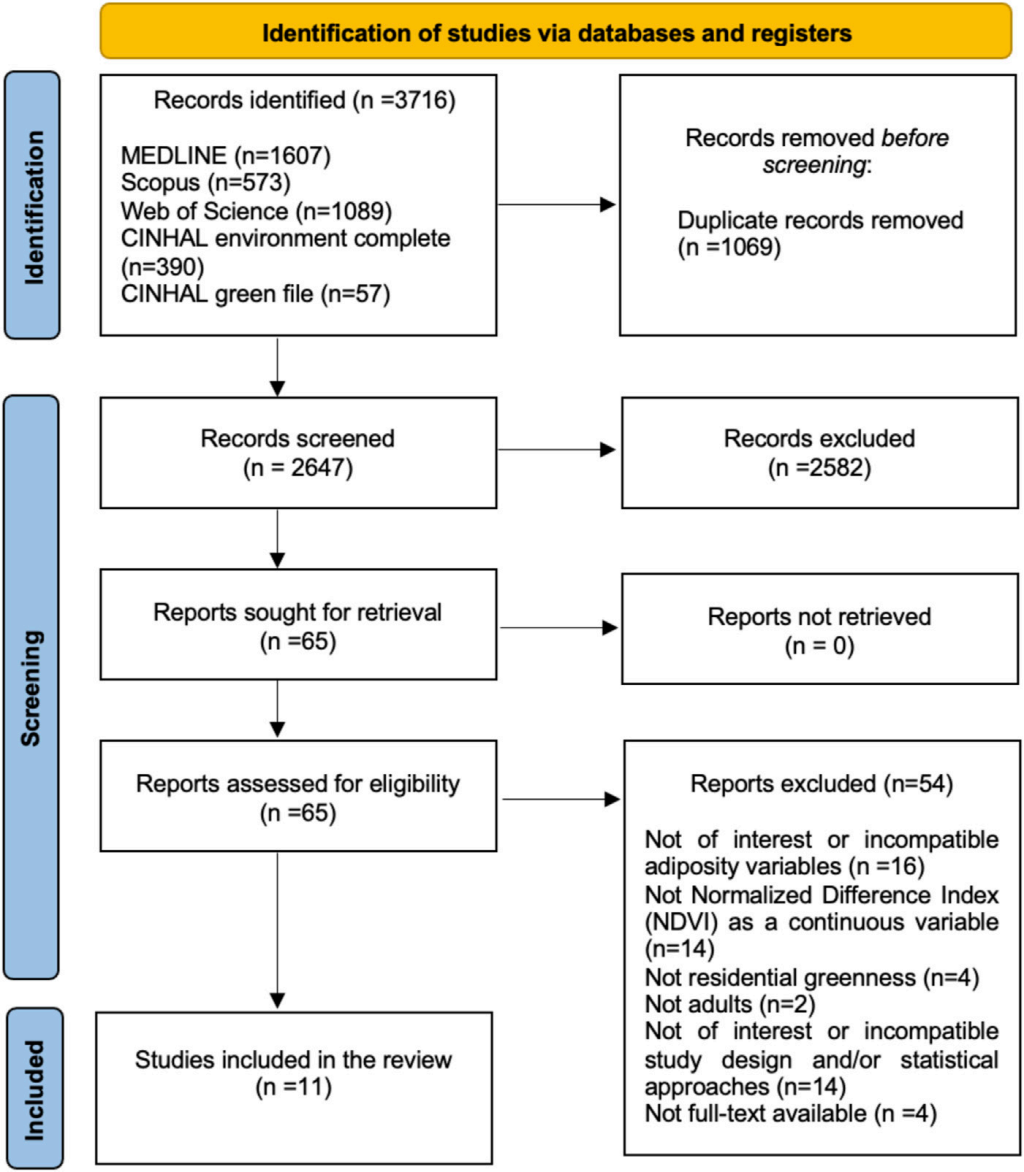
PRISMA flowchart of the stepwise assessment of articles obtained from the search strategy (worldwide, 2017–2023).

The systematic search identified a total of 3,716 published articles. Of these, 1,069 (29%) duplicate publications were removed, thus leaving 2,647 (71%) potentially eligible articles, which were extracted to review the title and abstract, following the inclusion and exclusion criteria. Of the 2,647 articles, 2,582 (98%) were excluded, with 65 (2%) articles finally selected for full-text reading to verify their eligibility. References to the excluded full-text articles are available in the Supplementary Material. Finally, 11 (17%) studies [50–57, 60–62] were included in this systematic review and meta-analysis (Figure 1).

The characteristics of each of the studies are detailed in Table 1; Supplementary Material. All studies were cross-sectional and published between 2020 and 2023. Seven studies addressed the waist circumference measured in centimeters (cm) [51, 52, 55, 56, 60–62], and five addressed blood lipids measured in millimoles per liter (mmol/L) [50, 52–54, 57]. Regarding the independent variable, residential greenness, all studies quantified it objectively through the NDVI index. The NVDI increment units and cut-off points for determining the adverse adiposity and blood lipid parameters applied by each study are detailed in Table 1. Of these studies, one was done in Canada [61], one in Spain [62], one in the UK [56], and the other eight studies in China [50–55, 57, 60]. The study samples ranged from 2,354 to 333,183 people, with a total of 564,254 individuals. All cross-sectional studies were done in general adult populations with a mean age of 55.95 years old. All studies included both sexes with one study considered the most women percentage resulting in 65.4% [50]. Additionally, all studies were conducted in urban settings, except two [51, 57] that were performed in rural ones.

**TABLE 1:** Characteristics of the cross-sectional studies included in the systematic review and meta-analysis (worldwide, 2017–2023).

Author	Country	Sample size (n, % female)	Age (mean)	BMI (mean, (kg/m^2^)	NDVI exposure definition^a^	NDVI increment unit	NDVI (mean or median)	Outcome	Outcome criteria	Odds ratio (95% CI)	Covariate adjustments
Fan et al. [50]	China	4.735 (65.4)	47.5	25.00	100 m 300 m 500 m 1000 m	0.20 0.11 0.10 0.08	0.45 0.46 0.47 0.47	Total cholesterol (mmol/L) Triglycerides (mmol/L) HDL-C (mmol/L) LDL-C (mmol/L)	Elevated total cholesterol: ≥6.22 mmol/L Elevated triglycerides: ≥2.26 mmol/L Reduced HDL-C: <1.04 mmol/L Elevated LDL-C: ≥4.14 mmol/L	Elevated TC 1.00 (0.65, 1.56) 1.16 (0.85, 1.59) 1.27 (0.91, 1.78) 1.21 (0.90, 1.63) Elevated TG 0.93 (0.79, 1.09) 0.91 (0.82, 1.02) 0.96 (0.86, 1.07) 0.97 (0.88, 1.07) Reduced HDL-C 0.87 (0.77, 0.98) 0.94 (0.87, 1.03) 0.97 (0.89, 1.05) 0.99 (0.91, 1.06) Elevated LDL-C 0.88 (0.68, 1.15) 0.95 (0.80, 1.14) 0.93 (0.78, 1.11) 0.97 (0.82, 1.14)	Age, sex, marital status, education levels
Fan et al. [51]	China	4.651 (65.10)	47.46	25.34	100 m 300 m 500 m 1000 m	0.20 0.12 0.09 0.07	0.47 0.48 0.47 0.47	Waist circumference (cm)	Abdominal obesity: WC > 102 cm in men and >88 cm in women	1.00 (0.86, 1.16) 0.92 (0.82, 1.03) 0.92 (0.84, 1.01) 0.98 (0.90, 1.06)	Age, sex, education levels, and marital status
Huang et al. [60]	China	24.845 (49.0)	45.6	24.4	500 m 1000 m	0.17 0.15	0.33 0.34	Waist circumference (cm)	Abdominal obesity: WC > 102 cm in men and >88 cm in women	0.88 (0.83, 0.93) 0.89 (0.84, 0.95)	Age, gender, ethnicity, and household income
Jiang et al. [57]	China	39.057 (60.66)	55.6	24.80	1000 m	0.09	0.52	Total cholesterol (mmol/L Triglycerides (mmol/L) LDL-C (mmol/L) HDL-C (mmol/L)	Elevated total cholesterol: ≥6.2 mmol/L Elevated Triglycerides: ≥2.3 mmol/L Elevated: LDL-C ≥4.1 mmol/L Reduced HDL-C: <1.0 mmol/L	1.05 (0.96, 1.14) 0.97 (0.91, 1.03) 0.94 (0.88, 1.00) 1.33 (1.21, 1.46)	Age, sex, matrimony, educational level, monthly income, cigarette use, alcohol intake, high-fat intake, adequate vegetables/fruits consumption, physical exercise, family history of dyslipidemia, BMI, and PM_2_
Li et al. [52]	China	38.288 (52.8)	53.63	NA	250 m 500 m 1000 m	NA	0.51 0.54 0.57	Waist circumference (cm) Triglycerides (mmol/L) HDL-C (mmol/L)	Abdominal obesity: WC ≥ 90 cm for men and ≥85 cm for women Elevated Triglycerides: ≥1.7 mmol/L Reduced HDL-C: <1.03 mmol/L in men and <1.29 mmol/L in women	Abdominal obesity 0.70 (0.52, 0.96) 0.66 (0.48, 0.91) 0.59 (0.43, 0.82) Elevated TG 0.93 (0.90, 0.97) 0.93 (0.89, 0.98) 0.98 (0.93, 1.02) Reduced HDL-C 1.05 (1.00, 1.10) 1.04 (0.99, 1.09) 1.00 (0.95, 1.05)	Age, sex, ethnicity, education, occupation, marital status, residential location, diet, smoking status, alcohol drinking status, temperature, and humidity
Nichani et al. [61]	Canada	14.550 (61.0)	55.2	27.40	400 m	NA	NA	Waist circumference (cm)	Abdominal obesity: WC ≥ 94 cm in men and ≥80 cm in women	0.96 (0.92, 1.00)	Age, sex, self-reported general health, current marital status, number of children in household, highest education level, current employment status, annual household income, and current smoking status
O’Callaghan-Gordo et al. [62]	Spain	2.354 (63.2)	61	23.45	300 m	0.09	0.23	Waist-to-hip ratio	Abdominal obesity: WHR was ≥0.85 for women and ≥0.90 for men	0.68 (0.45–1.01)	Age, level of education, socioeconomic status, area level economic status
Sarkar [56]	United Kingdom	333.183 (54.6)	56.5	27.47	500 m	0.24	0.16	Waist circumference (cm)	NA	0.67 (0.60, 0.73)	Individual-level covariates, SES and built environment exposures (retail density, street walkability, terrain, and PM_10_ and PM_2.5_
Wang et al. [54]	China	43.183 (55.4)	54.66	23.76	250 m 500 m 1,000 m	0.29 0.31 0.45	0.54 0.58 0.61	Total cholesterol (mmol/L Triglycerides (mmol/L) HDL-C (mmol/L) LDL-C (mmol/L)	Elevated total cholesterol: ≥6.22 mmol/L Elevated Triglycerides: ≥2.26 mmol/L Reduced HDL-C: <1.04 mmol/L Elevated LDL-C: ≥4.14 mmol/L	Elevated TC 0.97 (0.96, 0.99) 0.97 (0.96, 0.99) 0.97 (0.96, 0.99) Elevated TG 0.97 (0.95, 0.98) 0.96 (0.95, 0.98) 0.97 (0.95, 0.98) Reduced HDL-C 0.97 (0.95, 0.98) 0.96 (0.95, 0.98) 0.99 (0.97, 1.01) Elevated LDL-C 0.92 (0.90, 0.94) 0.92 (0.91, 0.94) 0.88 (0.86, 0.89)	Age, sex, ethnic, education level, occupation, marriage, insurance, smoking status, second-hand smoking, alcohol drinking status, resident, dietary intake, temperature, and humidity
Xiao et al. [55]	China	24.845 (49.0)	45.6	24.40	800 m 1000 m 1500 m	0.17 0.14 0.15	0.23 0.23 0.24	Waist circumference (cm)	Abdominal obesity: WC ≥ 102 cm for men and ≥88 cm for women	0.88 (0.82, 0.94) 0.89 (0.84, 0.95) 0.91 (0.86, 0.97)	Age, sex, ethnicity, education, income, smoking status, alcohol consumption and regular exercise
Xu et al. [53]	China	34.563 (55.30)	71.09	23.73	250 m 500 m 1250 m	0.12 0.11 0.11	0.32 0.32 0.34	Total cholesterol (mmol/L) Triglycerides (mmol/L) LDL-C (mmol/L) HDL-C (mmol/L)	Elevated total cholesterol: ≥6.22 mmol/L Elevated triglycerides: ≥2.26 mmol/L Reduced HDL-C: <1.04 mmol/L Elevated LDL-C: ≥4.14 mmol/L	Elevated TC 0.99 (0.97, 1.03) 1.01 (0.98, 1.04) 1.01 (0.98, 1.04) Elevated TG 0.94 (0.91, 0.97) 0.95 (0.92, 0.98) 0.97 (0.94, 0.99) Reduced HDL-C 0.96 (0.94, 0.99) 0.97 (0.94, 0.99) 0.98 (0.95, 1.00) Elevated LDL-C 0.99 (0.96, 1.03) 0.99 (0.96, 1.03) 0.97 (0.94, 1.00)	Age, gender, household registration, education, marital status, smoking, drinking, physical activity, BMI, WC, SBP, DBP

Abbreviations: BMI, body mass index; HDL-C, high-density lipoprotein cholesterol; LDL-C, low-density lipoprotein cholesterol; NA, not available; NDVI, normalized difference vegetation index; SD, standard deviation; TC, total cholesterol; TG, triglycerides; WC, waist circumference WHR, waist-to-hip ratio.

^a^ The zone of residential proximity to surrounding green space that was considered an exposure.

### Quality Assessment

The methodological quality for studies examining waist circumference and residential greenness was classified as good in 18% and as fair in 82% of the included studies (Table 2). Concerning both exposure variables, it was possible to ascertain that the primary reason for a fair methodological quality was the follow-up items.

**TABLE 2:** Methodological quality assessment with the Observational Cohort and Cross-Sectional Studies Tool from the National Institute of Health National Heart, Lung, and Blood Institute [46] (worldwide, 2017–2023).

Author	1	2	3	4	5	6	7	8	9	10	11	12	13	14	Score	Quality
Fan et al. [50]	Y	Y	Y	Y	Y	Y	N	Y	Y	N	Y	N	NA	Y	10	Fair
Fan et al. [51]	Y	Y	Y	Y	Y	Y	N	Y	Y	N	Y	N	NA	Y	10	Fair
Huang et al. [60]	Y	Y	Y	Y	Y	Y	N	Y	Y	N	Y	N	NA	Y	10	Fair
Jiang et al. [57]	Y	Y	Y	Y	Y	Y	N	Y	Y	N	Y	N	NA	Y	10	Fair
Li et al. [52]	Y	Y	Y	Y	Y	Y	N	Y	Y	N	Y	N	NA	Y	10	Fair
Nichani et al. [61]	Y	Y	Y	Y	Y	Y	N	N	Y	Y	Y	N	NA	Y	10	Fair
O’Callaghan-Gordo et al. [62]	Y	Y	Y	Y	Y	Y	N	N	Y	N	Y	N	NA	Y	9	Fair
Sarkar [56]	Y	Y	Y	Y	Y	Y	N	N	Y	N	Y	N	NA	Y	9	Fair
Wang et al. [54]	Y	Y	Y	Y	Y	Y	N	Y	Y	N	Y	N	NA	Y	10	Fair
Xiao et al. [55]	Y	Y	Y	Y	Y	Y	N	Y	Y	Y	Y	N	NA	Y	11	Good
Xu et al. [53]	Y	Y	Y	Y	Y	Y	N	Y	Y	Y	Y	N	NA	Y	11	Good

Abbreviations: N, no; NA, not applicable, Y, yes.

Numbers represent the questions included in The Quality Assessment Tool for Observational Cohort and Cross-Sectional Studies: 1. Question clear? 2. Population clearly defined? 3. >50% participants? 4. Recruitment populations consistent? 5. ¿Sample size justified? 6. Exposure assessed prior to outcome? 7. Sufficient timeframe? 8. Different exposure levels? 9. Valid exposure? 10. Repeated exposure assessment? 11. Valid outcomes? 12. Outcome assessors blinded? 13. Loss to follow up <20%? 14. Confounders adjusted for? Each cross-sectional study was rated as good, fair, or poor according to the National Institutes of Health’s Quality Assessment Tool for Observational Cohort and Cross-Sectional quality rating guide.

### Association Between Residential Greenness and Abdominal Obesity

**FIGURE 2:**
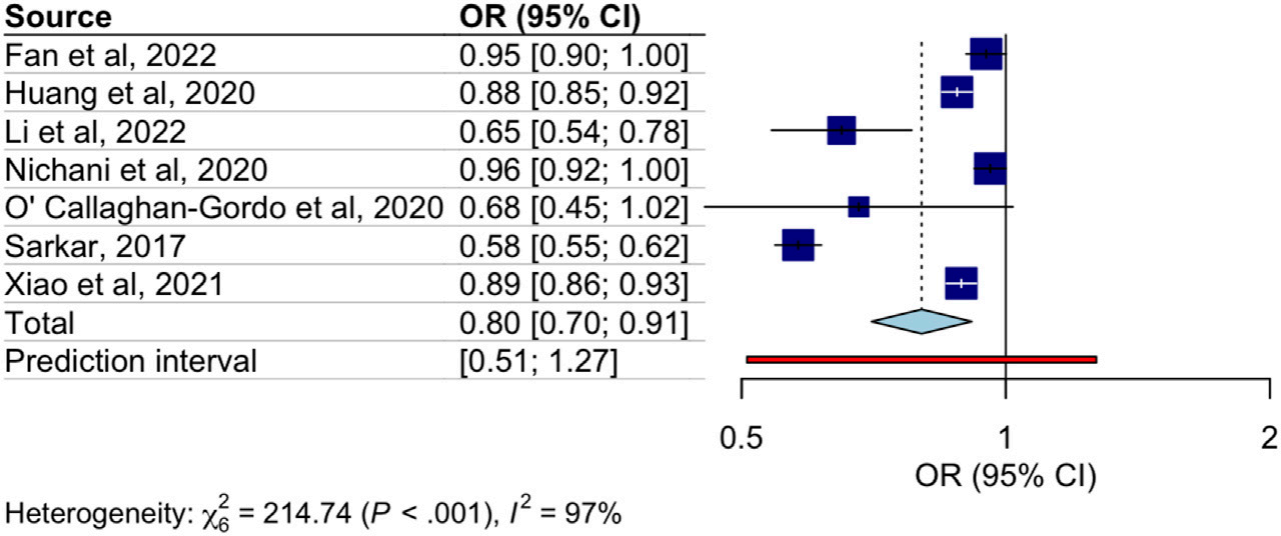
Association between increased Normalized Difference Vegetation Index values and the odds of abdominal obesity (worldwide, 2017–2022).

A significant inverse relationship was observed between an increase in surrounding greenness and reduced odds of abdominal obesity (OR: 0.80; 95% CI: 0.70–0.91; I^2^ = 85%) in a residential buffer zone of 1,500 m or less (Figure 2).

Subgroup analyses are displayed in Table 3. In the analysis conducted by sex [51, 55, 56, 60], the pooled ORs between higher NVDI values and the odds of abdominal obesity were significant only for females (OR: 0.82; 95% CI: 0.69–0.97; I^2^ = 95%) (Table 3). According to the socioeconomic status [55, 56, 60], the pooled ORs were significant for both individuals with lower (OR: 0.62; 95% CI: 0.41–0.93; I^2^ = 94%) and higher socioeconomic level (OR: 0.80; 95% CI: 0.66–0.97; I^2^ = 94%) (Table 3). Finally, according to the residential buffer zone subgroups (100 m [51, 52], 300 m [51, 62], 500 m [51, 52, 60], and 1,000 m [51, 55, 60]), the 500 m buffer zone (OR: 0.88; 95% CI: 0.81–0.96; I^2^ = 49%) and the 1000 m buffer zone (OR: 0.91; 95% CI: 0.86–0.97; I^2^ = 52%) showed a significant inverse relationship with the odds of abdominal obesity (Table 3).

**TABLE 3:** Subgroup analyses of the associations between residential greenness with central obesity and dyslipidemia in adults of all ages^a^ (worldwide, 2017–2023).

	Sex			Socioeconomic status			NDVI residential buffer zones		
	n	OR (95% CI)	I^2^ (%)	n	OR (95% CI)	I^2^ (%)	n	OR (95% CI)	I^2^ (%)
Abdominal obesity	4^b^ 4^c^	0.84 (0.58, 1.23) **0.82 (0.69, 0.97)**	**98.0** **95.0**	3^d^ 3^e^	0.62 (0.41, 0.93) 0.80 (0.66, 0.97)	**94.0** **94.0**	2^f^ 2^g^ 3^h^ 3^i^	0.78 (0.47, 1.31) 0.84 (0.65, 1.10) **0.88 (0.81, 0.96)** **0.91 (0.86, 0.97)**	**88.0** 50.0 49.0 52.0
Total cholesterol	3^b^ 3^c^	1.03 (1.00, 1.06) 1.04 (0.95, 1.14)	0.0 52.0	-			2^f^ 2^j^ 3^h^ 2^i^	1.05 (0.96, 1.14) **0.98 (0.96, 0.99)** 0.99 (0.95, 1.04) 1.03 (0.85, 1.25)	0.0 29.0 75.0 53.0
Triglycerides	3^b^ 3^c^	0.94 (0.85, 1.04) 0.96 (0.88, 1.06)	65.0 **73.0**	-			2^j^ 3^h^ 5^i^	**0.96 (0.93, 0.99)** **0.94 (0.92, 0.97**) **0.97 (0.96, 0.98)**	67.0 0.0 0.0
LDL-C	3^b^ 3^c^	1.14 (0.89, 1.47) 1.04 (0.80, 1.35)	**97.0** **94.0**	-			2^j^ 3^h^ 3^i^	0.95 (0.89, 1.02) 0.95 (0.89, 1.01) 1.04 (0.78, 1.40)	**92.0** **85.0** **97.0**
HDL-C	3^b^ 3^c^	0.92 (0.84, 1.00) **0.96 (0.93, 0.98)**	**71.0** 0.0	-			3^j^ 4^h^ 4^i^	0.99 (0.95, 1.02) 0.98 (0.95, 1.01) 0.99 (0.97, 1.00)	**82.0** **69.0** 0.0

^a^ Studies that did not report results stratified by sex or socioeconomic status were not included in the subgroup analysis.

^b^ Studies that reported male data.

^c^ Studies that reported female data.

^d^ Studies that reported a lower socioeconomic status.

^e^ Studies that reported a higher socioeconomic status.

^f^ Studies that reported 100 m NDVI information.

^g^ Studies that reported 300 m NDVI information.

^h^ Studies that reported 500 m NDVI information.

^i^ Studies that reported 1,000 m NDVI information.

^j^ Studies that reported 250 m NDVI information.

The bold front indicates statistical significative effects size. I^2^ informed heterogeneity (Bold text refers to statistically significant at p < 0.05). n represents the number of studies included in the subgroup analysis.

### Association of Dyslipidemia With Residential Greenness

**FIGURE 3:**
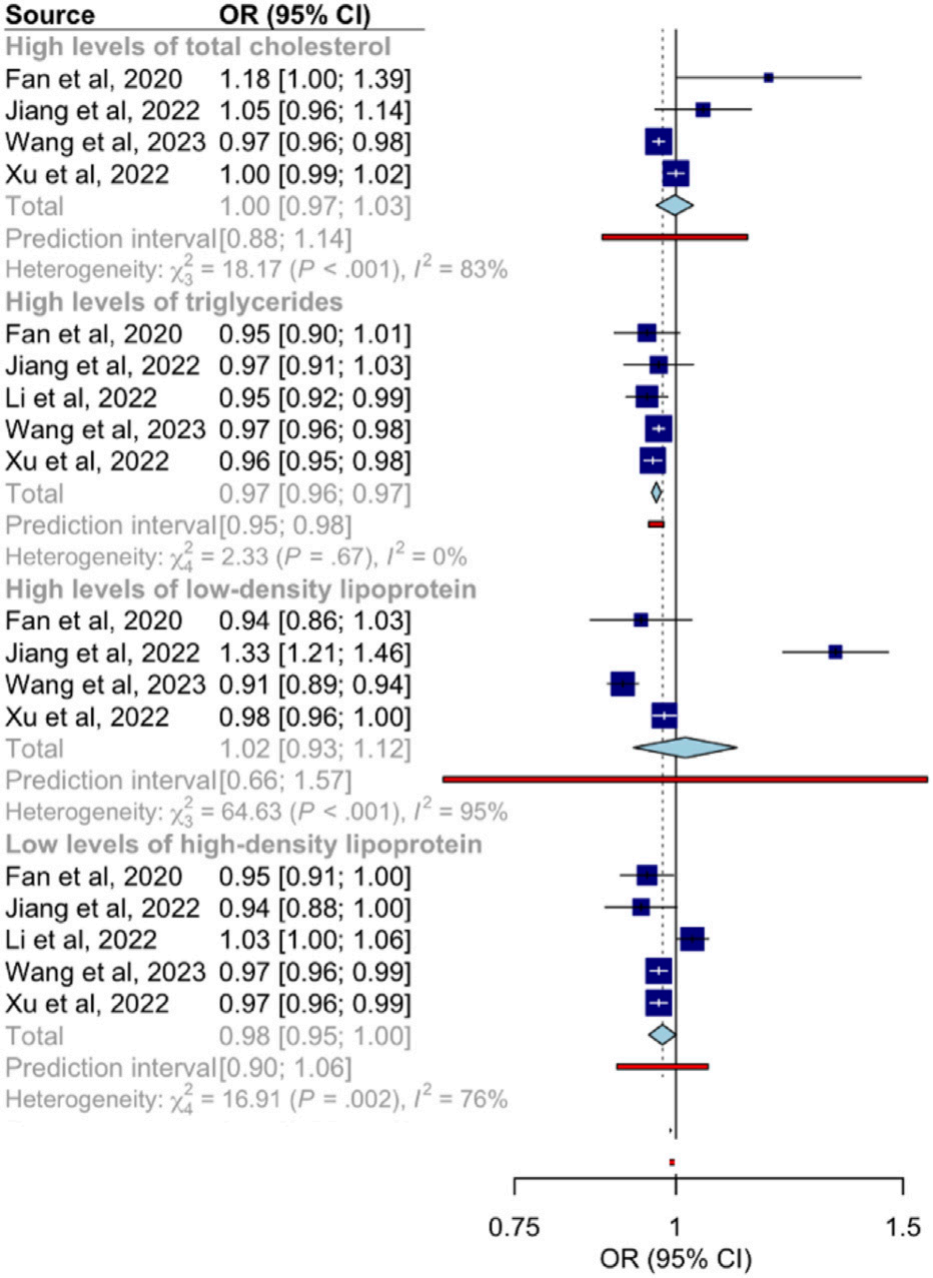
Associations between increased Normalized Difference Vegetation Index values and the odds of dyslipidemia (worldwide, 2020–2023).

A significant inverse relationship was observed between an increase in surrounding greenness and diminished odds of elevated triglycerides (OR: 0.97; 95% CI: 0.96–0.97; I^2^ = 0%) and low HDL-C levels (OR: 0.98; 95% CI: 0.95–1.00; I^2^ = 76%) in a residential buffer zone of 1,250 m or less (Figure 3). Regarding other blood lipids, no significant associations were observed between higher NVDI values and the odds of high levels of total cholesterol (OR: 1.00; 95% CI: 0.97–1.03; I^2^ = 83%) and LDL-C (OR: 1.02; 95% CI: 0.93–1.12; I^2^ = 95%). In the analysis conducted by sex [50, 53, 57], the pooled OR in the female category showed a significant reduction in the odds of low HDL-C levels (OR: 0.96; 95% CI: 0.93–0.98; I^2^ = 0%) (Table 3). Finally, the pooled estimates for the residential buffer zone subgroups (100 m [50, 57], 250 m [52–54], 500 m [50, 52–54], and 1,000 m [50, 52, 54, 57]) were similar to the main pooled ORs (Table 3).

## Discussion

This novel systematic review and meta-analysis provides a synthesis of the available evidence assessing the associations of residential greenness with abdominal obesity and dyslipidemia in the general adult population. Our findings suggest an inverse association between higher NDVI values and the odds of abdominal obesity, with a significant association observed in women. In addition, our results show significant inverse associations of higher NDVI values with the odds of elevated triglycerides levels and the odds of low HDL-C. A significant association in women was also observed for HDL-C levels.

The waist circumference is an important health indicator of abdominal obesity, and its reduction through regular physical activity and a healthy diet has been demonstrated in numerous studies [63–66]. On the other hand, dyslipidemia has been also associated with certain healthy behaviors and lifestyles, also it includes regular physical activity and a healthy diet low in saturated fats. Access to greenness has been found to encourage physical activity by providing attractive places for practice exercise [12, 67–69]. Healthy cities also incorporate not only green spaces but also promote walkability, which in turn leads to increased physical activity among citizens [7, 70, 71]. Furthermore, some studies have found a correlation between exercise and healthier dietary habits [72, 73]. However, further research is required to ascertain the relationship between greenery and diet-related behaviors.

A noteworthy finding of our study is the significant association between surrounding residential greenness and reduced triglyceride levels. The lipoprotein lipase is a pivotal enzyme in lipid metabolism, and its primary function is to catalyze the hydrolysis of triglycerides [74, 75]. This lipoprotein also facilitates the transfer of lipids to HDL-C particles, promoting their maturation and increasing their levels, which enhances the reverse cholesterol transport process. This relationship between lipoprotein lipase, triglycerides and HDL is essential for maintaining a healthy lipid profile and reducing the risk of cardiovascular disease [76, 77]. Residential greenness could be associated with higher levels of physical activity and a considerable body of research has shown a positive correlation between exercising and an increase in the activity of lipoprotein lipase [78–80]. In addition, physical activity exerts an indirect influence on triglyceride levels by improving insulin sensitivity [81, 82].

Concerning the results of the subgroup analysis, it was found that women benefited more from greenery than men. This may be due to certain gender roles, as women tend to walk more than men as a form of physical exercise [83]. It can be observed that women tend to prefer activities that are less intense and more accessible than those chosen by men, who tend to select higher-intensity exercises such as weightlifting [84]. Furthermore, women frequently assume roles requiring more physical activity throughout the day, such as childcare and household management [85]. This can result in an increased level of incidental physical activity including walking.

Although data on socio-economic status were only available for waist circumference, this finding is particularly noteworthy. In this study, significant associations were observed between higher NDVI values and reduced waist circumference in both socio-economic groups. Specifically, living near green spaces was associated with a 38% lower probability of increased waist circumference for individuals with low socio-economic status, and a 20% lower probability for those with high socio-economic status. The lower odds of abdominal obesity among people with low socio-economic status can be attributed to several factors. Firstly, individuals with a higher socio-economic status are more likely to reside in proximity to green spaces. Furthermore, these spaces are often of superior quality and are more likely to be well-maintained and equipped [18, 86]. Secondly, individuals with this condition are not only more health-conscious and knowledgeable but also have more free time and more flexible working hours [87, 88]. Nevertheless, the association between residential greenness and socio-economic status observed in this study was more pronounced among individuals with diminished socio-economic standing. Green spaces offer such as free recreational and physical exercise opportunities for those with fewer resources [89, 90]. Nevertheless, this greater benefit in this group may be influenced by the fact that people with lower socioeconomic status tend to walk more frequently due to factors such as the lack of private transport and the necessity to move around the city cost-effectively [91, 92].

A further noteworthy finding of this meta-analysis concerns the associations between NDVI values of the smaller residential buffer sizes (250 m, 500 m, and 1,000 m) and the analyzed adiposity and blood lipid parameters. This may be attributed to the enhanced accessibility of nearby greenery, which may be further motivated to regular physical activities such as walking, jogging, and exercise than those located further away from the domicile [16].

The present systematic review and meta-analysis has some limitations that should be addressed. Firstly, the included studies employed a cross-sectional design, as this is the most prevalent type in the extant literature on the subject. This may be attributable to the reduced complexity and cost of such studies in comparison to their longitudinal counterparts. Nevertheless, this methodological limitation imposes constraints on our capacity to establish causal relationships, and the potential for confounding factors or biases cannot be discounted. Secondly, most of the analyses showed a considerable heterogeneity since specific between-study differences such as age groups and buffer sizes. Thirdly, only studies that analyzed NDVI continuously were included to avoid heterogeneity due to arbitrary categorization, which may result in the exclusion of other potential studies. Fourthly, the restricted geographical scope of the studies can be considered as a further limitation. It is evident that a considerable proportion of the included studies are from China, thereby reflecting a geographical bias in the extant literature. While the findings are relevant within that specific context, their generalizability to other regions, which may possess different socio-economic, cultural, political and health conditions, can vary significantly between countries, and it may affect the way studies are developed and interpreted. Studies conducted in multiple countries may offer a more complete and robust perspective. Moreover, the exclusion of studies published in languages other than English or Spanish may introduce language bias, potentially omitting valuable findings from regions where publications in other languages are more common. Finally, due to the limited number of studies (n < 10) analyzing the study associations, meta-regressions [36] and publication bias [93] could not be performed.

Conversely, this study has several strengths that warrant mention. To our knowledge, this is the first meta-analysis to examine the relationships of residential greenness with abdominal obesity and blood lipid levels, notably the inclusion of a wide range of publication dates achieved by the absence of temporal restrictions in the search criteria. Furthermore, restricting the independent variable to NDVI has ensured objectivity and accuracy to the results, as it provides a quantitative, objective, and comparable measure of the available vegetation. In addition, the subgroup analyses by sex and socio-economic status, as conducted in this study, enables a more comprehensive and detailed results. Finally, the incorporation of studies classified as fair quality may introduce methodological biases that could influence the interpretation of the overall results. However, the inclusion of studies with varying qualities ensures a more comprehensive evaluation to determine the potential limitations of the existing body of evidence regarding the relationships between residential greenness, abdominal obesity and blood lipid levels.

The findings of this systematic review and meta-analysis underscore the critical role of residential greenery and urban vegetation in promoting public health. Integrating green spaces into urban planning and public health policies is essential to maximize health benefits and foster inclusive, accessible environments. The proximity of residential areas to urban parks could enhance levels of moderate and vigorous physical activity, thereby contributing to the reduction of obesity [94, 95]. Similarly, dense urban tree planting has been associated with lower mortality risk and improved health and wellbeing [96, 97]. Moreover, initiatives such as green corridors have been showed to enhance active mobility, thereby providing metabolic and cardiovascular health advantages [98, 99]. To maximize these benefits, it is vital to promote multidisciplinary collaborations between urban planners, public health professionals, and policymakers [100]. The provision of safe, appealing environments because of such efforts not only encourages healthier behaviors, but also contributes to reducing the burden of chronic diseases and the associated costs to health systems.

## Conclusion

The results of this study showed that increased residential greenness may be associated with reduced odds of both abdominal obesity and elevated triglycerides levels in the general adult population. This study provides a deeper understanding of how the green environment may be related to adiposity and blood lipid parameters in adults, as well as possible disparities according to specific sociodemographic characteristics. These findings could also contribute to reinforcing the importance of green spaces in public health, as this element, which is a fundamental component of healthy cities, could facilitate improved cardiovascular and metabolic health among the adult population by influencing their behavioral choices. Nevertheless, further longitudinal studies with greater geographical variability are required.

## Data Availability

The datasets generated or analyzed during the current study are not publicly available due to the research team’s decision. Still, they are available from the corresponding author on reasonable request.
