# Supplementary material for: Surrounding Residential Greenness and Health: Associations With Abdominal Obesity and Dyslipidemia. A Meta-Analysis of Cross-Sectional Studies
Source: Public Health Rev. 2025 Feb 18;46:1608163. doi: 10.3389/phrs.2025.1608163 (PMC11880791; doi:10.3389/phrs.2025.1608163)
Supplement: Supplementary file 1 [file Table1.DOCX]

**SUPPLEMENTARY MATERIAL**

S1: References of full text excluded articles.

1. NOT OF INTEREST OR INCOMPATIBLE ADIPOSITY VARIABLES^1–16^
2. NOT NORMALIZED DIFFERENCE INDEX (NDVI)AS A CONTINOUS VARIABLE^17–30^
3. NOT RESIDENTIAL GREENNESS^31–34^
4. NOT ADULTS^35,36^
5. NOT OF INTEREST OR INCOMPATIBLE STUDY DESIGN AND/OR STATISTICAL APPROACHES^37–50^
6. NOT FULL-TEXT AVAILABLE^51–54^

REFERENCES

1. Yang BY, Markevych I, Heinrich J, et al. Residential greenness and blood lipids in urban-dwelling adults: The 33 Communities Chinese Health Study. *Environ Pollut*. 2019;250:14-22. doi:10.1016/j.envpol.2019.03.128

2. Brown SC, Lombard J, Wang K, et al. Neighborhood Greenness and Chronic Health Conditions in Medicare Beneficiaries. *Am J Prev Med*. 2016;51(1):78-89. doi:10.1016/j.amepre.2016.02.008

3. Wang K, Lombard J, Rundek T, et al. Relationship of Neighborhood Greenness to Heart Disease in 249 405 US Medicare Beneficiaries. *J Am Heart Assoc*. 2019;8(6):e010258. doi:10.1161/JAHA.118.010258

4. Pan M, Liu F, Zhang K, et al. Independent and interactive associations between greenness and ambient pollutants on novel glycolipid metabolism biomarkers: A national repeated measurement study. *Environ Res*. 2023;233:116393. doi:10.1016/j.envres.2023.116393

5. Jiang Y, Wang S, Ren L, Yang L, Lu Y. Effects of built environment factors on obesity risk across three types of residential community in Beijing. *J Transp Health*. 2022;25:101382. doi:10.1016/j.jth.2022.101382

6. Yang L, Chan KL, Yuen JWM, et al. Effects of Urban Green Space on Cardiovascular and Respiratory Biomarkers in Chinese Adults: Panel Study Using Digital Tracking Devices. *JMIR Cardio*. 2021;5(2):e31316. doi:10.2196/31316

7. Duncan GE, Avery A, Hurvitz PM, Moudon AV, Tsang S, Turkheimer E. Cohort Profile: TWINS study of environment, lifestyle behaviours and health. *Int J Epidemiol*. 2019;48(4):1041-1041h. doi:10.1093/ije/dyy224

8. Jia X, Yu Y, Xia W, et al. Cardiovascular diseases in middle aged and older adults in China: the joint effects and mediation of different types of physical exercise and neighborhood greenness and walkability. *Environ Res*. 2018;167:175-183. doi:10.1016/j.envres.2018.07.003

9. Xiao Y, Liu C, Lei R, et al. Associations of PM2.5 composition and green space with metabolic syndrome in a Chinese essential hypertensive population. *Chemosphere*. 2023;343:140243. doi:10.1016/j.chemosphere.2023.140243

10. Yu W, Liu Z, La Y, et al. Associations between residential greenness and the predicted 10-year risk for atherosclerosis cardiovascular disease among Chinese adults. *Sci Total Environ*. 2023;868:161643. doi:10.1016/j.scitotenv.2023.161643

11. Ke P, Xu M, Xu J, et al. Association of residential greenness with the risk of metabolic syndrome in Chinese older adults: a longitudinal cohort study. *J Endocrinol Invest*. 2023;46(2):327-335. doi:10.1007/s40618-022-01904-5

12. Liu T, Cai B, Peng W, et al. Association of neighborhood greenness exposure with cardiovascular diseases and biomarkers. *Int J Hyg Environ Health*. 2021;234:113738. doi:10.1016/j.ijheh.2021.113738

13. Yang BY, Liu KK, Markevych I, et al. Association between residential greenness and metabolic syndrome in Chinese adults. *Environ Int*. 2020;135:105388. doi:10.1016/j.envint.2019.105388

14. Paoin K, Pharino C, Phosri A, et al. Association between greenness and cardiovascular risk factors: Results from a large cohort study in Thailand. *Environ Res*. 2023;220:115215. doi:10.1016/j.envres.2023.115215

15. Paquet C, Orschulok TP, Coffee NT, et al. Are accessibility and characteristics of public open spaces associated with a better cardiometabolic health? *Landsc Urban Plan*. 2013;118:70-78. doi:10.1016/j.landurbplan.2012.11.011

16. Torres Toda M, Miri M, Heydari H, et al. A study on exposure to greenspace during pregnancy and lipid profile in cord blood samples. *Environ Res*. 2022;214:113732. doi:10.1016/j.envres.2022.113732

17. Coombes E, Jones AP, Hillsdon M. The relationship of physical activity and overweight to objectively measured green space accessibility and use. *Soc Sci Med*. 2010;70(6):816-822. doi:10.1016/j.socscimed.2009.11.020

18. Leng H, Li S, Yan S, An X. Exploring the Relationship between Green Space in a Neighbourhood and Cardiovascular Health in the Winter City of China: A Study Using a Health Survey for Harbin. *Int J Environ Res Public Health*. 2020;17(2):513. doi:10.3390/ijerph17020513

19. Knobel P, Maneja R, Bartoll X, et al. Quality of urban green spaces influences residents’ use of these spaces, physical activity, and overweight/obesity. *Environ Pollut*. 2021;271:116393. doi:10.1016/j.envpol.2020.116393

20. Yu W, Li Y, Zhang K, et al. The association between early pregnancy exposure to green space and maternal glucolipid metabolism disorders: evaluation of the mediating role of serum 25-hydroxyvitamin D. *Environ Sci Pollut Res Int*. 2023;30(14):40978-40986. doi:10.1007/s11356-022-25073-3

21. Tharrey M, Klein O, Bohn T, Malisoux L, Perchoux C. Nine-year exposure to residential greenness and the risk of metabolic syndrome among Luxembourgish adults: A longitudinal analysis of the ORISCAV-Lux cohort study. *Health Place*. 2023;81:103020. doi:10.1016/j.healthplace.2023.103020

22. Figaroa MNS, Gielen M, Casas L, et al. Early-life residential green spaces and traffic exposure in association with young adult body composition: a longitudinal birth cohort study of twins. *Environ Health*. 2023;22(1):18. doi:10.1186/s12940-023-00964-1

23. Vrijheid M, Fossati S, Maitre L, et al. Early-Life Environmental Exposures and Childhood Obesity: An Exposome-Wide Approach. *Environ Health Perspect*. 2020;128(6):67009. doi:10.1289/EHP5975

24. Plans E, Gullón P, Cebrecos A, et al. Density of Green Spaces and Cardiovascular Risk Factors in the City of Madrid: The Heart Healthy Hoods Study. *Int J Environ Res Public Health*. 2019;16(24):4918. doi:10.3390/ijerph16244918

25. Rundle AG, Neckerman KM, Judd SE, et al. Cumulative Experience of Neighborhood Walkability and Change in Weight and Waist Circumference in REGARDS. *Am J Epidemiol*. 2023;192(12):1960-1970. doi:10.1093/aje/kwad134

26. Carroll SJ, Dale MJ, Taylor AW, Daniel M. Contributions of Multiple Built Environment Features to 10-Year Change in Body Mass Index and Waist Circumference in a South Australian Middle-Aged Cohort. *Int J Environ Res Public Health*. 2020;17(3):870. doi:10.3390/ijerph17030870

27. Daniel M, Carroll SJ, Niyonsenga T, Piggott EJ, Taylor A, Coffee NT. Concurrent assessment of urban environment and cardiometabolic risk over 10 years in a middle-aged population-based cohort. *Geogr Res*. 2019;57(1):98-110. doi:10.1111/1745-5871.12318

28. Gianaros PJ, Miller PL, Manuck SB, et al. Beyond Neighborhood Disadvantage: Local Resources, Green Space, Pollution, and Crime as Residential Community Correlates of Cardiovascular Risk and Brain Morphology in Midlife Adults. *Psychosom Med*. 2023;85(5):378-388. doi:10.1097/PSY.0000000000001199

29. Pan J, Hu K, Yu X, et al. Beneficial associations between outdoor visible greenness at the workplace and metabolic syndrome in Chinese adults. *Environ Int*. 2024;183:108327. doi:10.1016/j.envint.2023.108327

30. Fan S, Feng W, Zhou Z, et al. Association between residential greenness and overweight/obesity among rural adults in northwestern China. *Environ Res*. 2022;204(Pt D):112358. doi:10.1016/j.envres.2021.112358

31. Pereira G, Christian H, Foster S, et al. The association between neighborhood greenness and weight status: an observational study in Perth Western Australia. *Environ Health*. 2013;12(1):49. doi:10.1186/1476-069X-12-49

32. Bao WW, Yang BY, Zou ZY, et al. Greenness surrounding schools and adiposity in children and adolescents: Findings from a national population-based study in China. *Environ Res*. 2021;192:110289. doi:10.1016/j.envres.2020.110289

33. Yang H, He D, Lu Y, Ren C, Huang X. Disentangling residential self-selection from the influence of built environment characteristics on adiposity outcomes among undergraduate students in China. *Cities*. 2021;113:103165. doi:10.1016/j.cities.2021.103165

34. Hu LX, Fan S, Ma Y, et al. Associations between greenspace surrounding schools and lipid levels in Chinese children and teenagers. *Environ Pollut*. 2023;317:120746. doi:10.1016/j.envpol.2022.120746

35. Dadvand P, Villanueva CM, Font-Ribera L, et al. Risks and benefits of green spaces for children: a cross-sectional study of associations with sedentary behavior, obesity, asthma, and allergy. *Environ Health Perspect*. 2014;122(12):1329-1335. doi:10.1289/ehp.1308038

36. Markevych I, Standl M, Sugiri D, et al. Residential greenness and blood lipids in children: A longitudinal analysis in GINIplus and LISAplus. *Environ Res*. 2016;151:168-173. doi:10.1016/j.envres.2016.07.037

37. Persson Å, Pyko A, Lind T, et al. Urban residential greenness and adiposity: A cohort study in Stockholm County. *Environ Int*. 2018;121(Pt 1):832-841. doi:10.1016/j.envint.2018.10.009

38. Anza-Ramirez C, Lazo M, Zafra-Tanaka JH, et al. The urban built environment and adult BMI, obesity, and diabetes in Latin American cities. *Nat Commun*. 2022;13(1):7977. doi:10.1038/s41467-022-35648-w

39. Lei R, Zhang L, Liu X, et al. Residential greenspace and blood lipids in an essential hypertension population: Mediation through PM2.5 and chemical constituents. *Environ Res*. 2024;240:117418. doi:10.1016/j.envres.2023.117418

40. Zhou W, Wang Q, Kadier A, et al. The role of residential greenness levels, green land cover types and diversity in overweight/obesity among older adults: A cohort study. *Environ Res*. 2023;217:114854. doi:10.1016/j.envres.2022.114854

41. Daniels K, Lê-Scherban F, Auchincloss AH, et al. Longitudinal associations of neighborhood environment features with pediatric body mass index. *Health Place*. 2021;71:102656. doi:10.1016/j.healthplace.2021.102656

42. Almeida LFF, Barreto SM, Souza RCF de, Cardoso L de O, Giatti L. Neighborhood greenspace and cardiometabolic risk factors: Cross-sectional and longitudinal analysis in ELSA-Brasil participants. *Health Place*. 2021;72:102699. doi:10.1016/j.healthplace.2021.102699

43. de Keijzer C, Basagaña X, Tonne C, et al. Long-term exposure to greenspace and metabolic syndrome: A Whitehall II study. *Environ Pollut*. 2019;255:113231. doi:10.1016/j.envpol.2019.113231

44. Feng S, Meng Q, Guo B, et al. Joint exposure to air pollution, ambient temperature and residential greenness and their association with metabolic syndrome (MetS): A large population-based study among Chinese adults. *Environ Res*. 2022;214(Pt 1):113699. doi:10.1016/j.envres.2022.113699

45. Huang B, Liu Y, Chen Y, Wei H, Dong G, Helbich M. Establishing associations between residential greenness and markers of adiposity among middle-aged and older Chinese adults through multilevel structural equation models. *Int J Hyg Environ Health*. 2020;230:113606. doi:10.1016/j.ijheh.2020.113606

46. Paquet C, Coffee NT, Haren MT, et al. Food environment, walkability, and public open spaces are associated with incident development of cardio-metabolic risk factors in a biomedical cohort. *Health Place*. 2014;28:173-176. doi:10.1016/j.healthplace.2014.05.001

47. Jiang J, Xiang Z, Liu F, et al. Associations of residential greenness with obesity and BMI level among Chinese rural population: findings from the Henan Rural Cohort Study. *Environ Sci Pollut Res*. 2022;29(49):74294-74305. doi:10.1007/s11356-022-20268-0

48. Mei Y, Li A, Zhao J, et al. Association of Long-term exposure to air pollution and residential greenness with lipid profile: Mediating role of inflammation. *Ecotoxicol Environ Saf*. 2023;257:114920. doi:10.1016/j.ecoenv.2023.114920

49. Yang BY, Hu LW, Jalaludin B, et al. Association Between Residential Greenness, Cardiometabolic Disorders, and Cardiovascular Disease Among Adults in China. *JAMA Netw Open*. 2020;3(9):e2017507. doi:10.1001/jamanetworkopen.2020.17507

50. Guo J, Wu J, Wei D, et al. Association between greenness and dyslipidemia in patients with coronary heart disease: A proteomic approach. *Ecotoxicol Environ Saf*. 2022;231:113199. doi:10.1016/j.ecoenv.2022.113199

51. Ke P, Xu M, Xu J, et al. Association of residential greenness with the risk of metabolic syndrome in Chinese older adults: a longitudinal cohort study. *J Endocrinol Invest*. 2023;46(2):327-335. doi:10.1007/s40618-022-01904-5

52. Ye L, Zhou J, Tian Y, et al. Association of greenness exposure with waist circumference and central obesity in Chinese adults aged 65 years and over. *Zhonghua Yu Fang Yi Xue Za Zhi*. 2023;57:86-92. doi:10.3760/cma.j.cn112150-20221117-01118

53. Huang W, Xu S, Fang Q, Dong G. Association of greenness surrounding schools and homes with adiposity levels in children and adolescents. *Huan Jing Yu Zhi Ye Yi Xue J Environ Occup Med*. 2022;39(1):23-. doi:10.11836/JEOM21387

54. Zhao Y, Fang T, Du M, et al. Association of community built environment with obesity among elderly residents. *J Environ Occup Med*. Published online 2023:176-183.
